# Qualities required for new graduate visiting nurses: A qualitative study

**DOI:** 10.1002/jgf2.307

**Published:** 2020-03-16

**Authors:** Akiko Akiyama, Yumi Fukuyama

**Affiliations:** ^1^ Department of Nursing Faculty of Health Science Kio University Japan; ^2^ Faculty of Medicine Saga University Saga Japan

**Keywords:** home care, home‐visit nursing, Japan, visiting nurse

## Abstract

**Background:**

The qualities required for new graduates to become visiting nurses remain unclear. The present study aimed to clarify the qualities required for new graduate visiting nurses.

**Methods:**

Semi‐structured interviews were conducted with nine home‐visiting nurses. Content analysis was conducted by the transcribed data collected from visiting nurses.

**Results:**

Years of nursing experience were ranged from 16 to 50. In total, 23 attributes were extracted and categorized.

**Conclusion:**

Our findings indicated that the qualities required for new graduate visiting nurses were as follows: basic knowledge and skills required as professionals and appropriate attitudes as members of society.

## Graphical abstract

This study sought to clarify the qualities required for new graduate visiting nurses from the perspectives of established visiting nurses. The findings indicated that the qualities required for new graduate visiting nurses from the perspectives of visiting nurses were as follows: (a) basic knowledge and skills required as professionals and (b) appropriate attitudes as members of society.

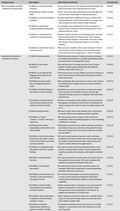

## 1 INTRODUCTION

Home‐visit nursing stations would play a crucial role in providing home‐based medical and end‐of‐life care.^1^ To retain the human resources needed to provide home‐based medical and end‐of‐life care, the National Association for Visiting Nurse Services plans to establish a nursing education program that allows new graduates to become visiting nurses and increases new graduate nurses working at home‐visit nursing stations.^2^, ^3^, ^4^ However, from the perspective of visiting nurses, the qualities required and whether it is possible for new graduates to become visiting nurses remain unclear. Therefore, the present study aimed to clarify the qualities required for new graduate visiting nurses from the perspectives of visiting nurses.

## 2 METHODS

### 2.1 Study design and period

Semi‐structured interviews were conducted with visiting nurses who worked at a home‐visit nursing station in Japan. The nurse station is a full member of the National Association for Visiting Nurse Services. The interviews were done between March and July 2018.

### 2.2 Research team and participants selection

Interviews were conducted by the two authors of this report. These authors have advanced experience as members of the New Graduate Visiting Nurse Review Committee at the prefecture level, are highly qualified academically, and are experienced in interviewing techniques. The research participants were selected by purposive selection; each of the participants in this research was related to this committee.

### 2.3 Interview guide

Each participant was interviewed according to a semi‐ structured interview guide consisting of one part. The guide was piloted and developed by the authors of this report. The interview began with open‐ended questions such as “What (do you think) are the essential qualities required for new graduate visiting nurses?” The participants were each asked to respond freely to questions, and their responses in turn informed further explorative questions. All interviews were recorded (with consent), transcribed, and referred back for validation.

### 2.4 Data analysis

The transcriptions of the interview were each analyzed qualitatively by thematic analysis, according to Braun & Clarke's^5^ six‐phase framework; raw data were coded, and then sorted into categories. Two themes were generated by collecting common codes and comparing and examining relationships between categories.

### 2.5 Ethical considerations

This study protocol was approved by the ethics committee at Kio University, Nara, Japan (approval number: H29‐24‐1). All participants in this study gave written informed consent.

## 3 RESULTS

The sample consisted of one man and eight women. Years of nursing experience ranged from 16 to 50 years (Table 1). In total, 23 attributes were extracted and categorized as follows: Basic knowledge and skills required as professionals and appropriate attitudes as members of society (Table 2).

**TABLE 1:** Participant characteristics

ID	Gender	Place of work	Types of legal entities	Official position	Duration of work (year)
A	Female	Kyushu	General Incorporated Association	Representative Directors at Incorporation	33
B	Female	Kyushu	General Incorporated Association	Chief Nurse	28
C	Female	Kyushu	Medical Institution	Chief Nurse	26
D	Female	Kyushu	Medical Institution	Chief Nurse	20
E	Female	Kyushu	Public Interest Incorporated Association	Director of District Support Center for Home Visit Nurse	40
F	Female	Kyushu	Public Interest Incorporated Association	Chief Nurse	22
G	Female	Kyushu	Public Interest Incorporated Association	Chief Nurse	20
H	Male	Shikoku	Medical Institution	Chief Nurse	16
I	Female	Chubu	Medical Institution	Chief Nurse	50

**TABLE 2:** Attributes and categories of the qualities required for new graduates working as visiting nurses

Category name	Subcategory	Code (Typical comment)	Comment (%)
Basic knowledge and skills required as professionals	(1) Ability to understand basic medicine	All you need to know is the anatomy and physiology that will be included in the national exam for nurse.	8 (88.9)
	(2) Experience in basic nursing skills	All you need is the basic experience to provide daily‐life care and basic knowledge of medical treatment.	7 (77.8)
	(3) Ability to assess the patient's condition	We also understand it difficult to assess a patient with multiple diseases. I think that it would be enough to be able to assess only the common major diseases.	5 (55.6)
	(4) Ability to understand evidence‐based nursing skills	It is enough to use techniques from the viewpoint of medical safety, to keep in mind the basic points of the national exam for nurse.	4 (44.4)
	(5) Ability to understand the contents of nursing practice	We don't need to be able to do anything which we have done during student‐nursing practice. Since various different things happen in each home‐care area, I think that young nurses should learn them in on‐the‐job training.	1 (11.1)
	(6) Ability to understand various social resources	When we were students, there were no lesson in home‐care nursing. Current students can learn about basic social resources at nursing school – which is enough.	1 (11.1)
Appropriate attitudes as members of society	(7) Ability to greet people	Patients become anxious if young nurse does not greet or introduce themselves in a socially appropriate proper way when they are visiting their home.	4 (44.4)
	(8) Ability to use proper honorifics	We need practice in nursing school on how to speak politely to build a good relationship with patients, families, other staff, and so on.	4 (44.4)
	(9) Ability to use appropriate language with patients and their families	I felt young nurses are too childish, and not yet mature to have correct manner talking with the patient and their family in practice.	4 (44.4)
	(10) Ability to social custom when visiting the patient's home	After graduating, they must learn so many social customs, for example how to align ones shoes neatly inside the entrance hall.	4 (44.4)
	(11) Ability to handle things in the patient's home carefully	Sometimes we need to move items to make some space to do our work in the patient's home, and they get annoyed if young nurses are not gentle with their items and furniture.	4 (44.4)
	(12) Ability to use the patient's home with permission of the patients and their families	We have to remember to ask politely if we use anything belonging to the patient in their home, for example we need to prepare hot water to clean the patient body, and so on.	3 (33.3)
	(13) Being well‐groomed	We have to wear clean smart clothes every visit and be so careful with correct makeup and hair condition.	3 (33.3)
	(14) Ability to “report,” “contact,” “consult” with one's supervisor	We want young nurses to report, note and discuss immediately when something happens or feel something is different or unusual.	2 (22.2)
	(15) Being honest while responding to the patients and their families	Being honest to tell us if you make a mistake, experience any failing or have any concerns at the patient's home, because in home‐care if you want to hide your mistake, you can hide it.	2 (22.2)
	(16) Ability to talk on the phone and find out the caller's name, contact information, and other necessary details	We need correct business manners while receiving a telephone call. There will be times when young nurses are unable to help the person who calls and they will need to transfer them to the right person.	2 (22.2)
	(17) Ability to sympathize with the patients and their families feelings	We need to spend a long time listening intently to the patients, hearing what are their concerns about their condition quietly with sympathy, with patient‐centered care.	2 (22.2)
	(18) Ability to read between the lines	We must write patient records with extra points that are not spoken in actual words. We need to capture the patient's perceptions of feeling living a life.	2 (22.2)
	(19) Ability to create basic documents	We have to know how to write a business letter. For example, initial and closing words, seasonal greetings. honorific expression, and so on.	1 (11.1)
	(20) Ability to smile warmly and maintain a calm expression in the patient's home	Non‐verbal communication can also be importance, as many nurses employ hand gestures when they communicate with patients without a shared language capability.	1 (11.1)
	(21) Ability to have friendly conversations with patients and their families	Verbal communication is importance to understand the patient's perspective of the illness and their needs. Young nurse can do this approach “ask‐tell‐ask” to patients and families in a friendly way.	1 (11.1)
	(22) Ability to listen attentively to patients' and their family members' stories	Home‐visiting nurse need to listen very carefully and try to get the key points being said (to record them later) when family members talk.	1 (11.1)
	(23) Ability to maintain relationships centered on patients and their families	The patient and family tend to welcome me as a family member or close friend, and I feel I have to act in the way they want.	1 (11.1)

## 4 DISCUSSION

We conducted the present study to clarify the qualities required for new graduate visiting nurses from the perspectives of visiting nurses. The study identified 23 attributes and two categories (basic knowledge and skills required as professionals and appropriate attitudes as members of society). There were several key findings.

### 4.1 Basic knowledge and skills required as professionals

Two components (ability to understand basic medicine and experience in basic nursing skills) were indicated by over the 70% of the participants. They seemed to be particularly important domains of the qualities required for new graduate nurses working at home‐visit nursing stations and were the basic skills necessary for nurses to obtain national qualifications. Findings suggest that it is important for new graduate visiting nurses to be able to assess patients’ lives including the use of social resources.

However, our previous study found that new graduate visiting nurses experienced difficulties with patients presenting complex multiple complications and comorbidities, as well as difficulties to understand and care based on the patient's needs^6^. It was suggested that new graduate visiting nurse was not considered to require high levels of proficiency in this study; the actual new graduates realized that high levels were needed in practice.

### 4.2 Appropriate attitudes as members of society

Seventeen common components of appropriate attitudes as members of society were identified. These components indicated key Japanese cultural factors. The results showed that many Japanese patients and their families preferred politeness. In addition, courtesies, including greetings, phraseology, deportment, appearance, and facial expressions, were very important elements of establishment of good relationship with patients and their families in Japan. Five common components of communication with patients and their families, “ability to sympathize with patients and their families feelings,” “ability to read between the lines,” “ability to have friendly conversations with patients and their families,” “ability to listen attentively to patients’ and their family members’ stories,” and “ability to maintain relationships centered on patients and their families,” were identified. Hirai et al emphasized the importance of trusting in the decision‐making process in Japanese end‐of‐life care.^7^ The current results also suggested that some Japanese patients and their families preferred to express themselves clearly in words, but many others tended to want visiting nurses to understand them without the need for conversation.

The study had several limitations. The number of participants was low and recruited from selected home‐visit nursing stations. However, previous studies^8^, ^9^, ^10^ have left unclear the qualities required for new graduate visiting nurses from the perspectives of visiting nurses who guidance and train to new graduates. Therefore, we believe that our findings provide a basis upon which to establish a nursing education program that enables new graduates to become home‐visiting nurses.

## 5 CONCLUSION

In conclusion, the findings indicated that the qualities required for new graduate visiting nurses from the perspectives of visiting nurses were as follows: (a) basic knowledge and skills required as professionals and (b) appropriate attitudes as members of society.

## CONFLICT OF INTEREST

The authors have stated explicitly that there are no conflicts of interest in connection with this article.
